# Supplementary material for: Preserving Information and Integrity in Cryo-EM: A Fourier-Space Deposition Approach
Source: bioRxiv. 2025 Nov 28:2025.11.26.690836. Preprint. [Version 1] doi: 10.1101/2025.11.26.690836 (PMC12676377; doi:10.1101/2025.11.26.690836)
Supplement: 1 [file NIHPP2025.11.26.690836V1-supplement-1.pdf]

bioRxiv preprint doi: https://doi.org/10.1101/2025.11.26.690836; this version posted November 28, 2025. The copyright holder for this preprint (which was not certified by peer review) is the author/funder, who has granted bioRxiv a license to display the preprint in perpetuity. It is made available under aCC-BY 4.0 International license.

Supplementary Materials

Case 1: Discussion about real space filtering

https://www.jiscmail.ac.uk/cgi-bin/wa-jisc.exe?A2=ind2508&L=CCPEM&P=34767

"I have rarely (never?) seen an unfiltered map deposited as the main map. The complication that Guillaume might have referred to here is that there are now machine-learning-driven software tools that produce improved maps (DeepEMhancer being one of them, but there are more and newer ones too - EMReady seems popular). The last time this issue was discussed, the majority expert opinion was that such "emhanced" maps should not be used for coordinate refinement. But they might be used to make figures, leading to the situation that the refinement map and figure map might not be the same map. This is not entirely new - we sometimes use different filtering resolution/sharpening levels to visualise poorly ordered peripheral regions in figures, which leads to a similar situation.

I believe that Guillaume's suggestion is to deposit the conventionally post-processed map as the main map (as this is usually/hopefully used to refine the coordinates) and the de-noised/enhanced/otherwise "manipulated" map as an additional map. At the risk of stating the obvious: I agree with this suggestion."

Case 2: Discussion about model to map cross correlation

https://www.jiscmail.ac.uk/cgi-bin/wa-jisc.exe?A2=ind2009&L=CCPEM&P=R12021

"The model vs. data CC is only 0.73, whereas the CC is 0.84 when I refine against the Relion-postprocessed map. CC is a function of sharpening in real space – any type of noise filtering outside the mask will improve CC, we should use masked data to see whether enhanced map (anything past Wiener filter and done in real space, including later AI maps) produces better refinement results. – Model to Map CC is a good measurement for unenhanced map quality, but not a good measure for model quality. Improvement in CC does not always indicate improvement in model.

bioRxiv preprint doi: https://doi.org/10.1101/2025.11.26.690836; this version posted November 28, 2025. The copyright holder for this preprint (which was not certified by peer review) is the author/funder, who has granted bioRxiv a license to display the preprint in perpetuity. It is made available under aCC-BY 4.0 International license.

Map enhancement does not provide additional information over the original reciprocal space information, but makes the map in real space more interpretable. Not just the EMhancer maps, but any real space maps provided by cryoEM software.

Reciprocal space has structural factor and sigma --- reciprocal space to real space map is a set of procedures involve variable weighting (sharpening). One can create a blend of the sharpening for different regions in real space to create an enhanced real space map. Such sharpening has no impact on refinement in reciprocal space because it is absorbed in weights (but will have impact in real space refinement)."

These two discussions show community concerns relevant to the issues presented in the main text.

Due to Fourier localization theorem, flattening a molecular map outside the mask does not affect the refined result, except for statistics based on correlation coefficient (CC) between the model and the map. Therefore, improvements in CC must be interpreted with caution. An increase in CC for a filtered map can occur without any real improvement in the model, for example, by filtering noise outside the molecular model. Additionally, more complex real space procedures modifying maps resulting from cryo-EM SPR may have features of the model building. For instance, alpha helical density can be modified to follow more closely an ideal alpha helix. This can again increase the CC between the modified map and the model, without any improvement in the refinement target. However, it has large value in visualization and initial model building. For this reason, any additional real-space-modified maps used during model building or inspection should also be deposited, and their deposition should be mandatory. These post-processed maps should be used only for visualization, not as targets in refinement.
